# Association between cognitive frailty and falls among older community dwellers in China: A Chinese longitudinal healthy longevity survey-based study

**DOI:** 10.3389/fnagi.2022.1048961

**Published:** 2023-01-13

**Authors:** Huihe Chen, Lanhui Huang, Wei Xiang, Yu Liu, Jian-Wen Xu

**Affiliations:** ^1^Department of Rehabilitation Medicine, The First Affiliated Hospital of Guangxi Medical University, Nanning, Guangxi, China; ^2^Department of Geriatric Endocrinology and Metabolism, The First Affiliated Hospital of Guangxi Medical University, Nanning, Guangxi, China; ^3^Department of Cardiology, Beijing Tsinghua Changgung Hospital, School of Clinical Medicine, Tsinghua University, Beijing, China; ^4^Department of Cardiology, The People’s Hospital of Guangxi Zhuang Autonomous Region, Nanning, Guangxi, China

**Keywords:** cognitive frailty, older adults, risk factor, fall, prediction model

## Abstract

**Background:**

The combined effect of cognitive impairment (CoI) and frailty on falls is controversial. This study aimed to explore whether older adults with cognitive frailty (*CF*) were at a higher risk of falls than those with only CoI or frailty and to present a fall prediction model based on *CF.*

**Methods:**

A total of 4,067 adults aged ≥ 60 years were included from the Chinese Longitudinal Healthy Longevity Survey through face-to-face interviews. Cognitive function and frailty were assessed using the mini-mental state examination scale and frailty index, respectively. Logistic regression was used to determine fall-associated risk factors and develop a fall prediction model. A nomogram was then plotted. The model performance was evaluated using the area under the curve (AUC), concordance index (C-index), and calibration curve. All analyses were performed using SPSS and R statistical packages.

**Results:**

The prevalence of *CF* and falls were 1.4 and 19.4%, respectively. After adjusting for covariates, the odds ratio of *CF*, frailty only, and CoI only for falls were 2.27 (95% CI: 1.29–3.97), 1.41 (95% CI: 1.16–1.73), and 0.99 (95% CI: 0.43–2.29), respectively. *CF*, sex, age, hearing difficulty, depression, anxiety, disability in instrumental activities of daily living, and serious illness in the past 2 years were independently associated with falls. A prediction model based on these factors yielded an AUC of 0.646 and a C-index of 0.641.

**Conclusion:**

Cognitive frailty (*CF*) exerted a cumulative effect on falls than did CoI or frailty alone. Joint assessments of cognitive function and frailty status may be beneficial for fall risk screening in community. A prediction model using *CF* as a factor could be helpful for this process.

## Introduction

Falls are a global public health concern (Florence et al., 2018). They can cause serious injuries, leading to disabilities and lethal complications. Particularly, as the global population is aging, the number of falls among older adults remains high (Moreland et al., 2020). According to available data, global fall prevalence ranges from 15 to 30% (Ganz and Latham, 2020; Han et al., 2021), which inevitably poses a huge burden on the medical system (Florence et al., 2018; Haddad et al., 2019). Hence, initiatives in fall detection and prevention are much needed.

An effective way to prevent falls is to identify risk factors and take precautions. So far, several factors associated with falls have been recognized, including sociodemographic characteristics (Tsai et al., 2020; Lee Y. Y. et al., 2021), functional decline (Moreira et al., 2018; Ogliari et al., 2021; Nagarkar and Kulkarni, 2022), psychological disorders (Luo et al., 2022), and medical conditions (Immonen et al., 2020; Lee S. et al., 2021). Among them, frailty is one of the most well-accepted factors. It is a geriatric syndrome and represents increased vulnerability to a multisystemic decline. A frail status increases the risk of adverse outcomes, especially falls (Rockwood et al., 2007; Clegg et al., 2013). Reliable assessments of frailty most often include frailty phenotype (FP; Fried et al., 2001) and frailty index (FI; Searle et al., 2008), although the main focus of these two parameters are not identical (Cesari et al., 2014). For example, components of FI include various deficits encompassing chronic conditions, psychological statuses, and symptoms. However, FP focuses more on the pre-disability syndrome. Researchers found that FI screened more people as frail regarding early detection (Blodgett et al., 2015). In many studies, an FI score greater than 0.21 is often defined as frailty (Gordon et al., 2021).

Another important fall risk factor is cognitive impairment (CoI). It is defined as a cognitive function worse than an individual’s expected level (Gauthier et al., 2006). A growing body of evidence has demonstrated that older adults with CoI are significantly at higher risk of falls (Muir et al., 2012; Montero-Odasso and Speechley, 2018). The mini-mental state examination (MMSE) and the revised Hasegawa Dementia (HDS-R) scales have proved to be reliable for screening for CoI in community setting (Senda et al., 2020). Since frailty and CoI often co-occur in aged individuals, relationship between cognitive frailty (*CF*; Kelaiditi et al., 2013) and falls has been explored. Reportedly, nearly 40% of older adults with *CF* experienced falls (Guo et al., 2022), and their risk of falling was at least twice as those without *CF* (Brigola et al., 2019; Kim et al., 2019a; Ma et al., 2021). However, whether *CF* exerts a cumulative effect on falls compared with CoI or frailty solely remains controversial. In community participants from Malaysia and the United States, *CF* showed a greater effect on fall risk than did CoI or frailty alone (Ge et al., 2021; Rivan et al., 2021). In contrast, Ma et al. (2021) observed that Chinese frail seniors had higher fall risk than those with *CF* compared with robust adults. Moreover, the extent to which *CF* could help in fall risk discrimination remains unknown. To fill this knowledge gap, we analyzed data from the Chinese Longitudinal Healthy Longevity Survey (CLHLS) to: (1) determine the prevalence of falls and *CF* in community dwellers aged 60 years and older, (2) explore whether *CF* increased fall risk compared with only CoI or frailty, and (3) develop a prediction model with *CF* for falls and validate the model’s performance.

## Materials and methods

### Design and population

**Figure 1:**
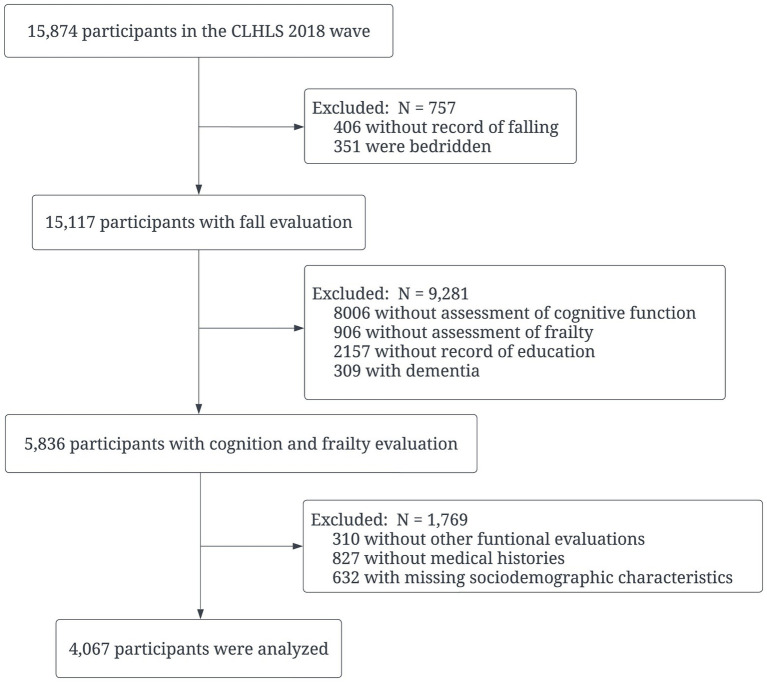
Flow chart of participants selection. CLHLS: Chinese Longitudinal Healthy Longevity Survey.

This cross-sectional study was conducted with participants included in the CLHLS 2018 wave. The CLHLS, conducting *via* face-to-face interviews, is one of the largest national longitudinal studies in the world for investigating healthy aging. In brief, the CLHLS covers 23 of 31 provinces in China, and the 2018 wave consisted of 15,874 respondents who were senior community dwellers. More details on the designs and objectives of the CLHLS have been reported elsewhere (Zeng et al., 2008). The inclusion criteria were: participants aged 60 years and above without missing dependent or independent variables. Those who were bedridden or diagnosed with dementia were excluded from the study. Finally, a total of 4,067 participants’ data were analyzed (Figure 1). Since the CLHLS was approved by the Ethical Committee of Peking University and all participants signed written consent, the current study was exempted from further ethical approval.

### Falls

A fall event was determined by the question “Have you fallen in the past year?” A “Yes” response was coded as a positive outcome, and the participant was categorized into the fall group; a “No” response represented a negative outcome, and the participants was categorized into the non-fall group as reference.

### Cognitive impairment, frailty, and cognitive frailty

Cognitive function was assessed using the MMSE scale. Domains of orientation, naming, attention, calculation, memory, and language were estimated by 24 items, with a total score of 30. As the MMSE score is influenced by education, years of schooling were taken into account. According to a previous study (Ma et al., 2017), CoI was defined as: (1) illiteracy level and MMSE score ≤ 17, (2) elementary school level and MMSE score ≤ 20, (3) middle school level and MMSE score ≤ 22, and (4) higher education level and MMSE score ≤ 24.

Frailty was evaluated using the FI following a standard procedure (Chen et al., 2020). A total of 35 dichotomous health deficits, including functional status, comorbidities, and physical activity were analyzed (Supplementary material). Each item was measured as with (1)/without (0) this deficit. For deficits with more than two categories, new values were constructed from 0 to 1. For example, the categories of self-reported health were recoded as follows: very good = 0, good = 0.25, average = 0.5, bad = 0.75, and very bad = 1. If participants had had more than one serious illness in the last 2 years, two scores were assigned to this deficit. The final FI was calculated as the unweighted sum of the existing deficits divided by the total number of possible deficits. In this study, only the FI with less than 30% missing values was calculated. According to the previous cutoff value (Gordon et al., 2021), frailty status was defined as FI > 0.21, and non-frail status was defined as FI ≤ 0.21.

Participants were then divided into four groups: (1) robust group: participants without CoI and FI ≤ 0.21, set as a reference in regression analyses, (2) CoI only group: participants with CoI and FI ≤ 0.21, (3) frailty only group: participants with FI > 0.21 but without CoI, and (4) *CF* group: participants with CoI and FI > 0.21.

### Sociodemographic variables

In this study, sociodemographic characteristics included sex (male/female), age (years), marital status (currently married/others), residence (city/town/rural), co-residence (with household member/in an institution/alone), schooling (years), education (illiteracy/literacy), occupation (agriculture/non-agriculture), household yearly income (0–30,000 Yuan or 4,302 USD/more than 30,000 Yuan or 4,302 USD), smoking (none/current/past), alcohol consumption (none/current/past), exercise (none/current/past), sleep duration (0–6 h per day/7–8 h per day/more than 9 h per day), and body mass index (BMI, kg/m^2^).

### Disabilities

Basic and instrumental activities of daily living (BADL and IADL, respectively) were used to detect disabilities. BADL was assessed by the following: (1) feeding, (2) bathing, (3) dressing, (4) toileting, (5) indoor transferring, and (6) continence. IALD covered abilities of the following: (1) visiting neighbors, (2) shopping independently, (3) cooking independently, (4) washing clothes, (6) walking 1 km, (7) squatting and standing three times, and (8) taking public transportation. Each of the above items in both BADL and IADL was scored from 1 to 3 points on three responses: completely independent = 1 point, partially dependent = 2 points, and completely dependent = 3 points. The total BADL and IADL scores ranged from 6 to 18 and 8 to 24, respectively. A higher score represents worse physical status. Disability was defined when any item in BADL or IADL scored three points (Zhang et al., 2021). BADL disability and IADL disability were the percentage of disabilities identified by each evaluation tool.

### Psychological health

The 10-item measure of the center for epidemiological studies depression (CES-D) scale was used to assess whether participants had depression. Participant’s feelings and behaviors were checked by the CES-D scale and the frequencies were recorded. In the current study, there were five responses to each item: (1) always, (2) often, (3) sometimes, (4) seldom, and (5) rarely or never. Following a previous study (Björgvinsson et al., 2013), responses of (4, 5) were collapsed into one group. Then each item was scored on a three-point scale, either 0-, 1-, 2-, or 3-pionts. The total CES-D score ranges from 0 to 30, and depression was distinguished with a CES-D score ≥ 10 (Björgvinsson et al., 2013). The anxiety status was assessed using the general anxiety disorder-7 (GAD-7) scale with seven questions. The response options and scoring methods for each question were identical: (1) never = 0 point, (2) several days = 1 point, (3) more than half of the days = 2 points, and (4) almost every day = 3 points. A GAD-7 score no less than 8 was confirmed as anxious (Plummer et al., 2016). The depressive and anxious statuses represented the percentage of depression and anxiety assessed by CES-D and GAD-7, respectively.

### Sensory evaluation

Hearing and vision were evaluated through self-reporting. Hearing difficulty was assessed by the question: “Do you have any difficulty with your hearing?” An answer of “Yes” represented a hearing decline and an answer of “No” represented intact hearing. Visual difficulty was assessed by the question “Can you see the break in the circle?” A response of “can see and distinguish” was recorded as an intact visual function, whereas other two responses of “can see only” and “cannot see” were categorized as with visual impairment.

### Medical conditions

As older adults have a high probability of comorbidities, relevant information was collected through participant’s recall. Medical conditions including hypertension, heart disease, diabetes mellitus, cerebrovascular disease, respiratory disease, dyslipidemia, arthritis, and a diagnosis of serious illness in the past 2 years were checked. In each category, the answer of “Yes/No” was recorded.

### Statistical analysis

Descriptive analysis was used to describe the above variables. Continuous variables are shown as mean ± SD, and *t*-test was used. Count and percentage of categorical variables are displayed, and the chi-square test was performed. Three sets of logistic regression models were developed to analyze the association between CoI only/frailty only/*CF* and falls. In Model 1, age, sex, education, and BMI were adjusted for. In Model 2, lifestyle habits and functional status were further adjusted, including smoking, alcohol consumption, exercise, sleep duration, disabilities, psychological status, and hearing and visual difficulties. In Model 3, chronic conditions were adjusted for. Odd ratios (ORs) were estimated. Significantly associated variables in Model 3 were presented in a forest plot and used to develop a fall prediction model. Then, a nomogram of this model was drawn. Receiver operating characteristic (ROC) were utilized to evaluate the discrimination capability of these models. C-index and calibration curve were used to assess the model performance. All the above statistical analysis was performed using SPSS (version 23.0, BMI Corp., United States) and R (RStudio, Inc., version 2022.02.3) software. A two-sided *p* < 0.05 was considered statistically significant.

## Results

Overall characteristics are shown in Tables 1, 2. Of the 4,067 participants, the mean age was 78.9 ± 9.79 years, with a greater proportion of male participants (54.5%) than of female. The mean MMSE and FI scores for the total sample were 28.2 ± 2.7 and 0.18 ± 0.07, respectively. The prevalence of falls was 19.4% (790) in this study. Compared to participants in the non-fall group, those who were female, older, and slept less than 7 h were more likely to fall. The MMSE and FI scores among the fallers were 27.6 ± 3.2 and 0.20 ± 0.07, respectively, significantly worse than the 28.3 ± 2.5 and 0.18 ± 0.06 scores in the non-fallers. Moreover, the fall group had higher proportions of frailty only (39.2% vs. 23.3%) and *CF* (3.0% vs. 1.0%) than the non-fall group. Both groups shared similar proportions of those with CoI only. For other functional evaluations, significant differences were found regarding BADL, IADL, depression, anxiety, and hearing difficulty. Fallers were more likely to perform poorly in the above functions than non-fallers. In addition, fallers had higher proportion of serious illnesses in the past 2 years.

**Table 1:** Sociodemographic characteristics of participants.

*n*	Overall4,067	Non-fall group3,277	Fall group790	*p*
Sex [*n* (%)]^a^				
Female	1851 (45.5)	1,422 (43.4)	429 (54.3)	<0.001
Male	2,216 (54.5)	1855 (56.6)	361 (45.7)	
Age (mean ± SD, years)^a^	78.88 ± 9.79	78.44 ± 9.67	80.72 ± 10.06	<0.001
BMI (mean ± SD, kg/m^2^)	22.97 ± 3.57	23.05 ± 3.53	22.67 ± 3.70	0.008
Residence [*n* (%)]				
City	1,307 (32.1)	1,036 (31.6)	271 (34.3)	0.33
Rural	1,556 (38.3)	1,260 (38.4)	296 (37.5)	
Town	1,204 (29.6)	981 (29.9)	223 (28.2)	
Marital status [*n* (%)]				
Currently married	2,342 (57.6)	1933 (59.0)	409 (51.8)	<0.001
Others	1725 (42.4)	1,344 (41.0)	381 (48.2)	
Co-residence [*n* (%)]				
Alone	596 (14.7)	468 (14.3)	128 (16.2)	0.269
Institution	127 (3.1)	99 (3.0)	28 (3.5)	
With household	3,344 (82.2)	2,710 (82.7)	634 (80.3)	
Schooling (mean ± SD, years)	5.43 ± 4.79	5.51 ± 4.71	5.09 ± 5.12	0.024
Education [*n* (%)]				
Illiteracy	1,015 (25.0)	777 (23.7)	238 (30.1)	<0.001
Literacy	3,052 (75.0)	2,500 (76.3)	552 (69.9)	
Occupation [*n* (%)]				
Agriculture	2,101 (51.7)	1,696 (51.8)	405 (51.3)	0.836
Non-agriculture	1966 (48.3)	1,581 (48.2)	385 (48.7)	
Household yearly income [*n* (%)]				
< 30,000 Yuan or 4,302 USD	1863 (45.8)	1,497 (45.7)	366 (46.3)	0.773
≥ 30,000 Yuan or 4,302 USD	2,204 (54.2)	1780 (54.3)	424 (53.7)	
Smoking [*n* (%)]				
Current	773 (19.0)	645 (19.7)	128 (16.2)	0.006
Past	723 (17.8)	599 (18.3)	124 (15.7)	
Never	2,571 (63.2)	2033 (62.0)	538 (68.1)	
Alcohol consumption [*n* (%)]				
Current	771 (19.0)	640 (19.5)	131 (16.6)	0.135
Past	537 (13.2)	435 (13.3)	102 (12.9)	
Never	2,759 (67.8)	2,202 (67.2)	557 (70.5)	
Exercise [*n* (%)]				
Current	1822 (44.8)	1,483 (45.3)	339 (42.9)	0.112
Past	262 (6.4)	199 (6.1)	63 (8.0)	
Never	1983 (48.8)	1,595 (48.7)	388 (49.1)	
Sleep duration [*n* (%)]				
< 7 h	1,499 (36.9)	1,190 (36.3)	309 (39.1)	0.038
7 ~ 8 h	1,697 (41.7)	1,399 (42.7)	298 (37.7)	
≥ 9 h	871 (21.4)	688 (21.0)	183 (23.2)	

BMI: body mass index.

a Factors significantly associated with falls in multivariable analysis.

**Table 2:** Functional status and medical history between groups.

n	Overall*n* = 4,067	Non-fall group*n* = 3,277	Fall group*n* = 790	*p*
Assessments of cognition and frailty				
MMSE (mean ± SD)	28.2 ± 2.7	28.3 ± 2.5	27.6 ± 3.2	<0.001
FI (mean ± SD)	0.18 ± 0.07	0.18 ± 0.06	0.20 ± 0.07	<0.001
Cognitive and frailty status				
Robust [*n* (%)]	2,953 (72.6)	2,480 (75.7)	473 (59.9)	<0.001
CoI only [*n* (%)]	40 (1.0)	33 (1.0)	7 (0.9)	0.757
Frailty only [*n* (%)]	1,016 (25.0)	730 (22.3)	286 (36.2)	<0.001
*CF* [*n* (%)]	58 (1.4)	34 (1.0)	24 (3.0)	<0.001
Other functional evaluations				
BADL (mean ± SD)	6.2 ± 1.0	6.2 ± 0.9	6.4 ± 1.2	<0.001
BADL disability [*n* (%)]	210 (5.2)	147 (4.5)	63 (8.0)	<0.001
IADL (mean ± SD)	10.5 ± 4.2	10.2 ± 3.9	11.6 ± 4.8	<0.001
IADL disability [*n* (%)]^a^	1,008 (24.8)	728 (22.2)	280 (35.4)	<0.001
CES-D (mean ± SD)	6.9 ± 3.7	6.7 ± 3.5	7.6 ± 4.0	<0.001
Depressive status [*n* (%)]^a^	804 (19.8)	595 (18.2)	209 (26.5)	<0.001
GAD-7 (mean ± SD)	1.2 ± 2.5	1.1 ± 2.4	1.8 ± 3.0	<0.001
Anxious status [*n* (%)]^a^	107 (2.6)	70 (2.1)	37 (4.7)	<0.001
With hearing difficulty [*n* (%)]^a^	950 (23.4)	709 (21.6)	241 (30.5)	<0.001
With vision difficulty [*n* (%)]	712 (17.5)	558 (17.0)	154 (19.5)	0.113
Medical history				
With serious illness* [*n* (%)]^a^	970 (23.9)	701 (21.4)	269 (34.1)	<0.001
With hypertension [*n* (%)]	1726 (42.4)	1,394 (42.5)	332 (42.0)	0.824
With DM (%)	448 (11.0)	365 (11.1)	83 (10.5)	0.656
With heart disease [*n* (%)]	782 (19.2)	625 (19.1)	157 (19.9)	0.644
With CVD (%)	498 (12.2)	399 (12.2)	99 (12.5)	0.831
With respiratory disease [*n* (%)]	437 (10.7)	347 (10.6)	90 (11.4)	0.555
With hyperlipidemia [*n* (%)]	249 (6.1)	196 (6.0)	53 (6.7)	0.494
With arthritis [*n* (%)]	485 (11.9)	381 (11.6)	104 (13.2)	0.256

MMSE: mini-mental state exam; FI: frailty index; CoI: cognitive impairment; CF: cognitive frailty; BADL: basic activities of daily living; IADL: instrumental activities of daily living; CES-D: epidemiological studies depression scale; GAD-7: General anxiety disorder-7; DM: diabetes mellitus. CVD: cerebrovascular disease.

a Factors significantly associated with falls in multivariable analysis.

* Serious illness in the past 2 years.

**Figure 2:**
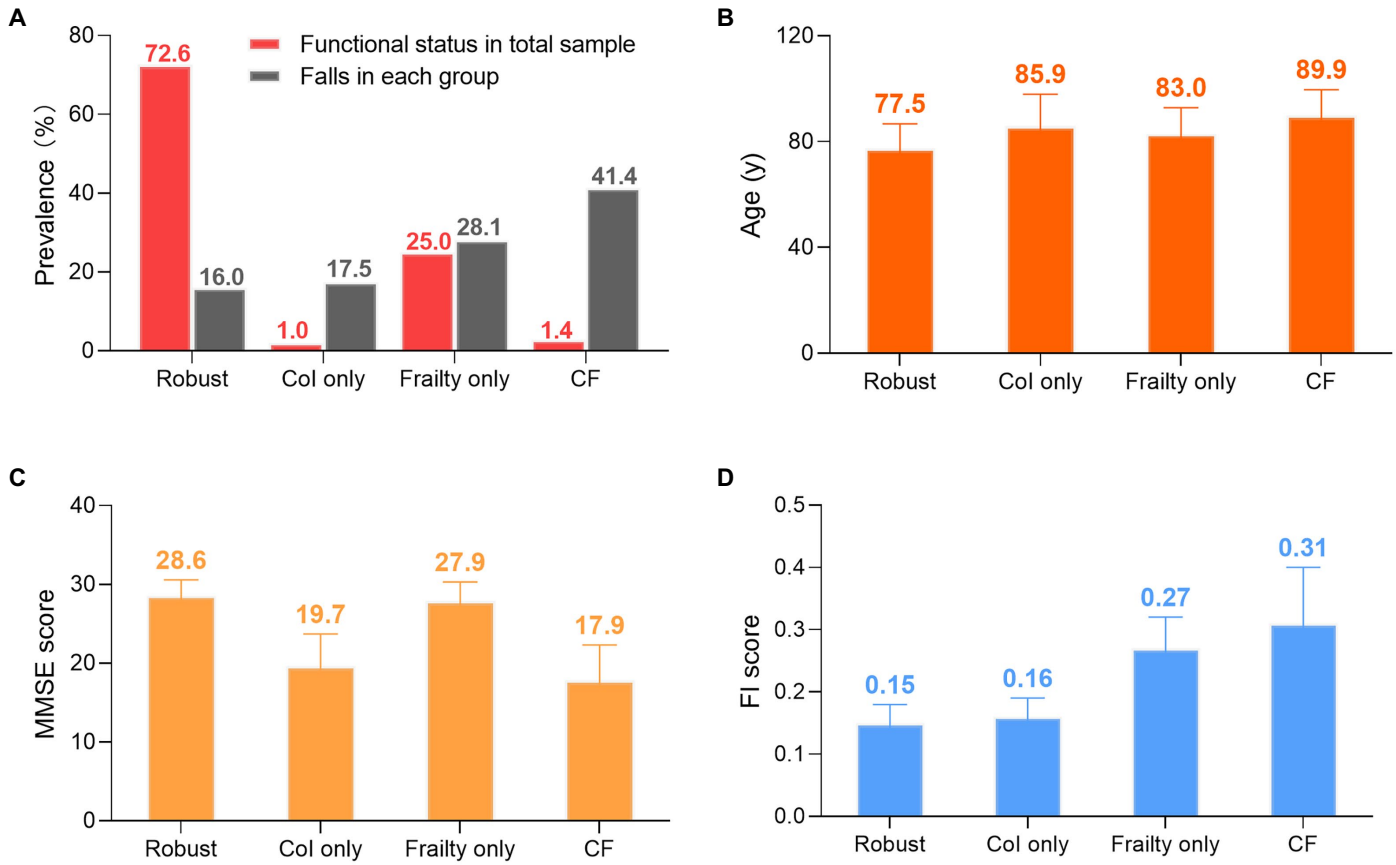
Groups differences. **(A)** Prevalence of functional statuses and falls. Red column represents number of specific status divided by 4,067. Gray column represents number of fallers in each group devided by the group’s total number. **(B)** Age: mean ± SD. **(C)** MMSE score: mean ± SD. **(D)** FI score: mean ± SD Col: cognitive impairment. *CF*: cognitive fraity. MMSE: mini-mental state examination. FI: fraity index. SD: standard deviation.

As shown in Figure 2, the prevalence of each status group was: 1.4% (58) in *CF* group, 1.0% (40) in CoI only group, 25.0% (1016) in frailty only group, and 72.6% (2953) in robust group. Fallers consisted of 41.4, 17.5, 28.2, and 16.0% in the *CF*, CoI only, frailty, and robust groups, respectively. Participants with *CF* tended to be male, older, and with poor cognitive decline and frail status among groups. The mean MMSE scores in each group from the worst to the best were: 17.7 ± 4.5, 19.7 ± 4.1, 27.9 ± 2.4, and 28.6 ± 2.0 in the *CF*, CoI only, frailty only, and robust groups, respectively, indicating that the categorization of CoI among our sample was mild to moderate. However, the pattern for mean FI was slightly different: 0.31 ± 0.09, 0.27 ± 0.05, 0.16 ± 0.03, and 0.15 ± 0.03 in the *CF*, frailty only, CoI only, and robust groups, respectively.

The results of regression analyses are shown in Table 3. Relationships between CoI only/frailty only/*CF* and falls remained unchanged from Model 1 to Model 3. Taking the robust group as the reference, older adults with *CF* were significantly at higher risk of falling than those with frailty only, with ORs of 3.50 vs. 1.93 in Model 1; 2.59 vs. 1.68 in Model 2; and 2.27 vs. 1.41 in Model 3, respectively (all *p* values < 0.05). The results from Model 3 suggested that compared with the robust group, participants with *CF* were 2.27 times more likely to fall, and the risk of falling for the frail only participants was 1.41 times, both with statistical significance. However, no significant associations were found between CoI only and falls, the OR (95%CI) in each model was: 1.02 (0.45–2.33) in Model 1; 0.96 (0.42, 2.21) in Model 2; 0.99 (0.43, 2.29) in Model 3.

**Table 3:** Associations between cognitive and/or frailty status with falls.

	Model 1	*p*	Model 2	*p*	Model 3	*p*
	OR (95%CI)		OR (95%CI)		OR (95%CI)	
Robust	Reference		Reference		Reference	
CoI only	1.02 (0.45, 2.33)	0.968	0.96 (0.42, 2.21)	0.919	0.99 (0.43, 2.29)	0.988
Frailty only	1.93 (1.63, 2.29)	<0.001	1.68 (1.40, 2.02)	<0.001	1.41 (1.16, 1.73)	0.001
*CF*	3.50 (2.04, 6.00)	<0.001	2.59 (1.49, 4.52)	0.01	2.27 (1.29, 3.97)	0.004

CoI: cognitive impairment; CF: cognitive frailty. OR: odds ratio. Model 1: adjusted for age, sex, residence, education, and BMI. Model 2: in addition to Model 1, smoking, alcohol consumption, exercise, sleep hours, disabilities, psychological status and hearing and visual difficulties were adjusted. Model 3: hypertension, heart disease, diabetes mellites, cerebrovascular disease, respiratory disease, dyslipidemia, arthritis, and diagnosed serious illness in the past 2 years were further adjusted.

**Figure 3:**
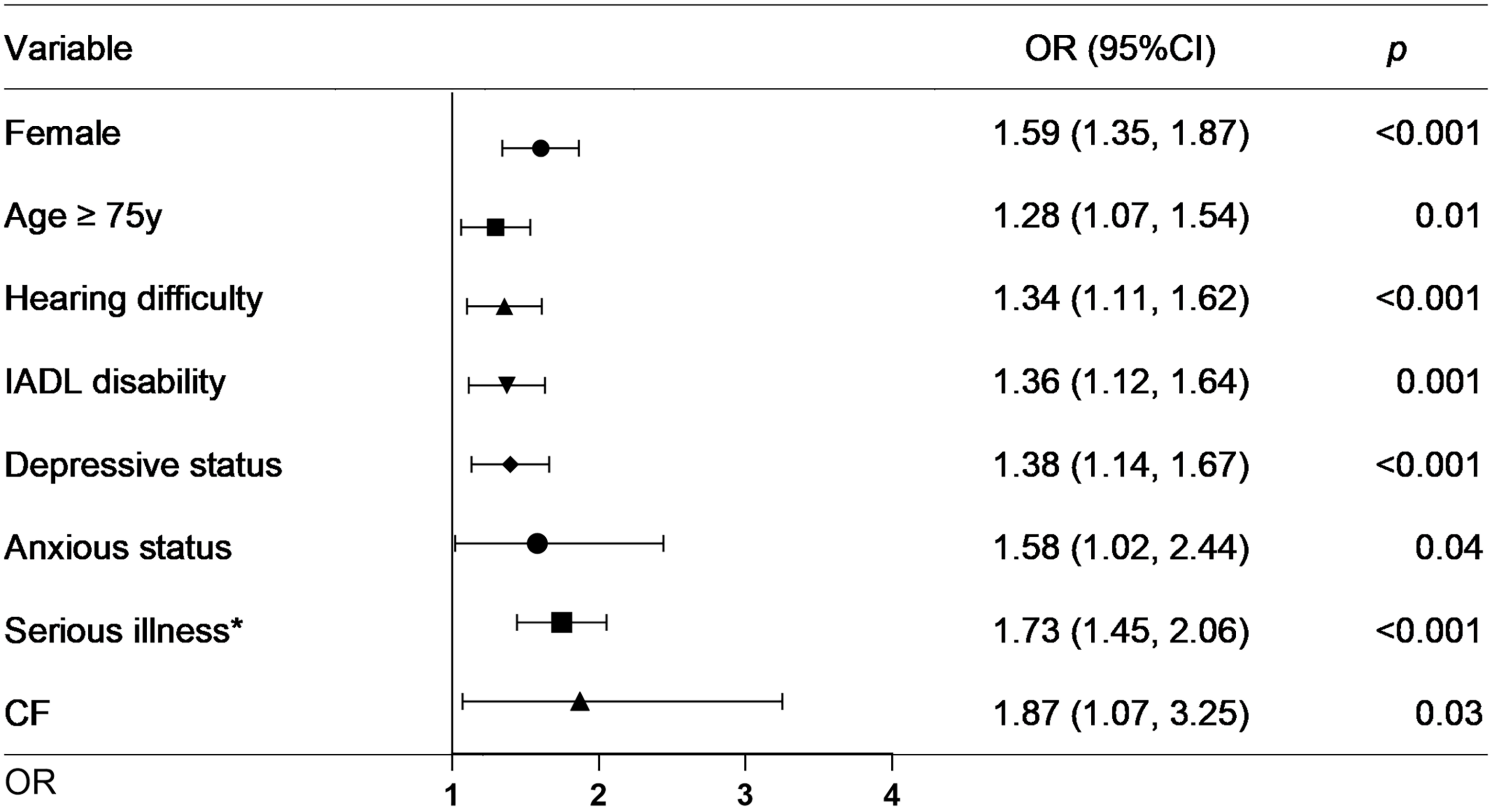
Forest plot for factors significantly associated with falls. IADL: instrumental activities of daily living. *CF*: cognitive fraity. OR: odds ratio. CI: confidence interval. ^*^Serious illness in the past 2 years.

**Figure 4:**
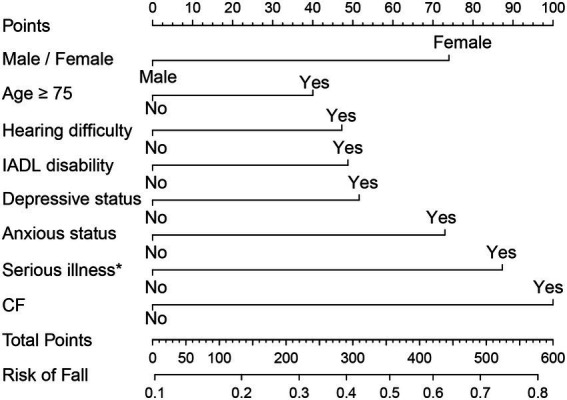
The normogram for fall prediction in community dwellers with CF. IADL: instrumental activities of daily living. *CF*: cognitive fraity. ^*^Serious illness in the past 2 years.

**Figure 5:**
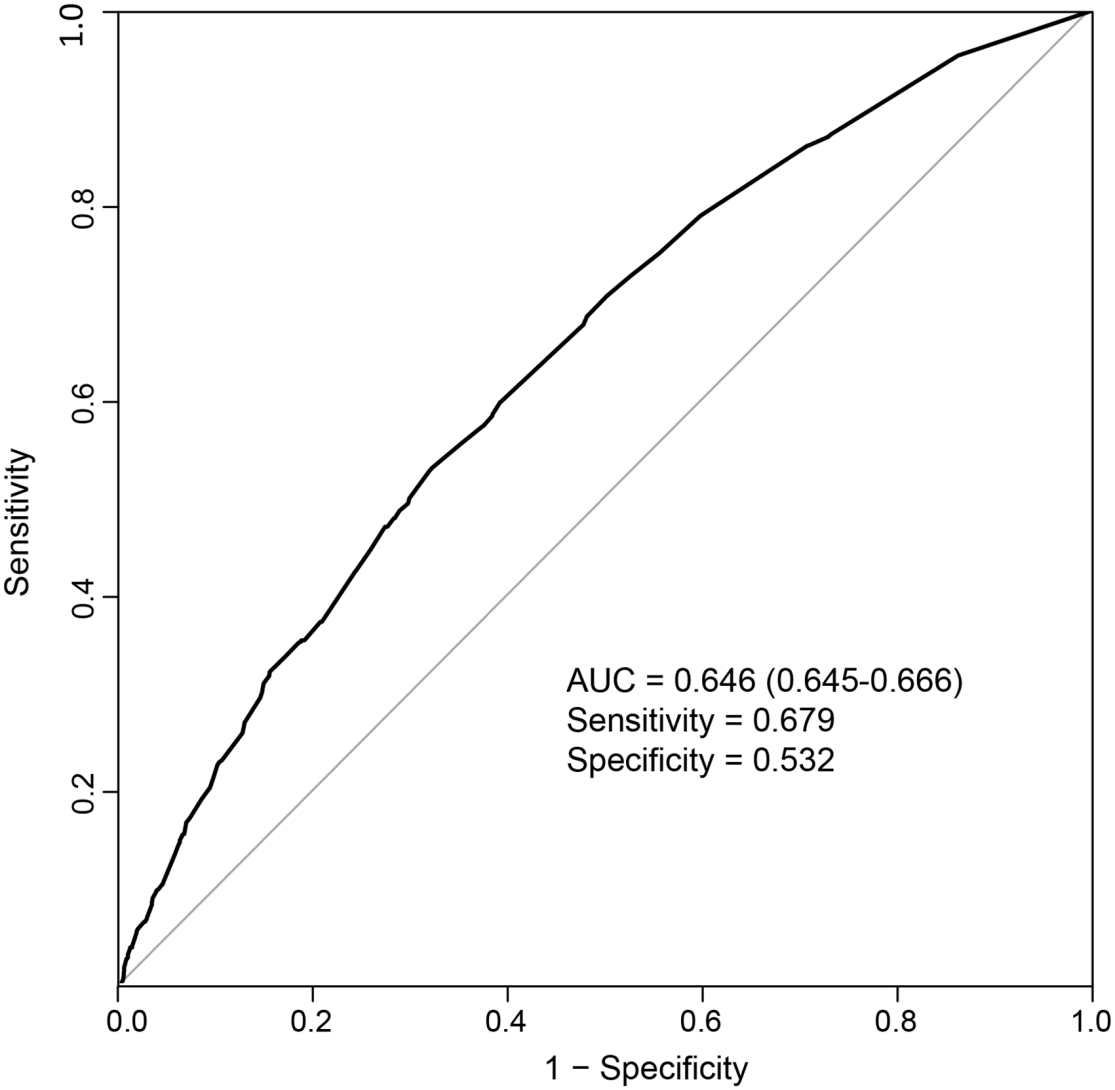
Receiver operating characteristic curve for the prediction model. AUC: area under the curve.

**Figure 6:**
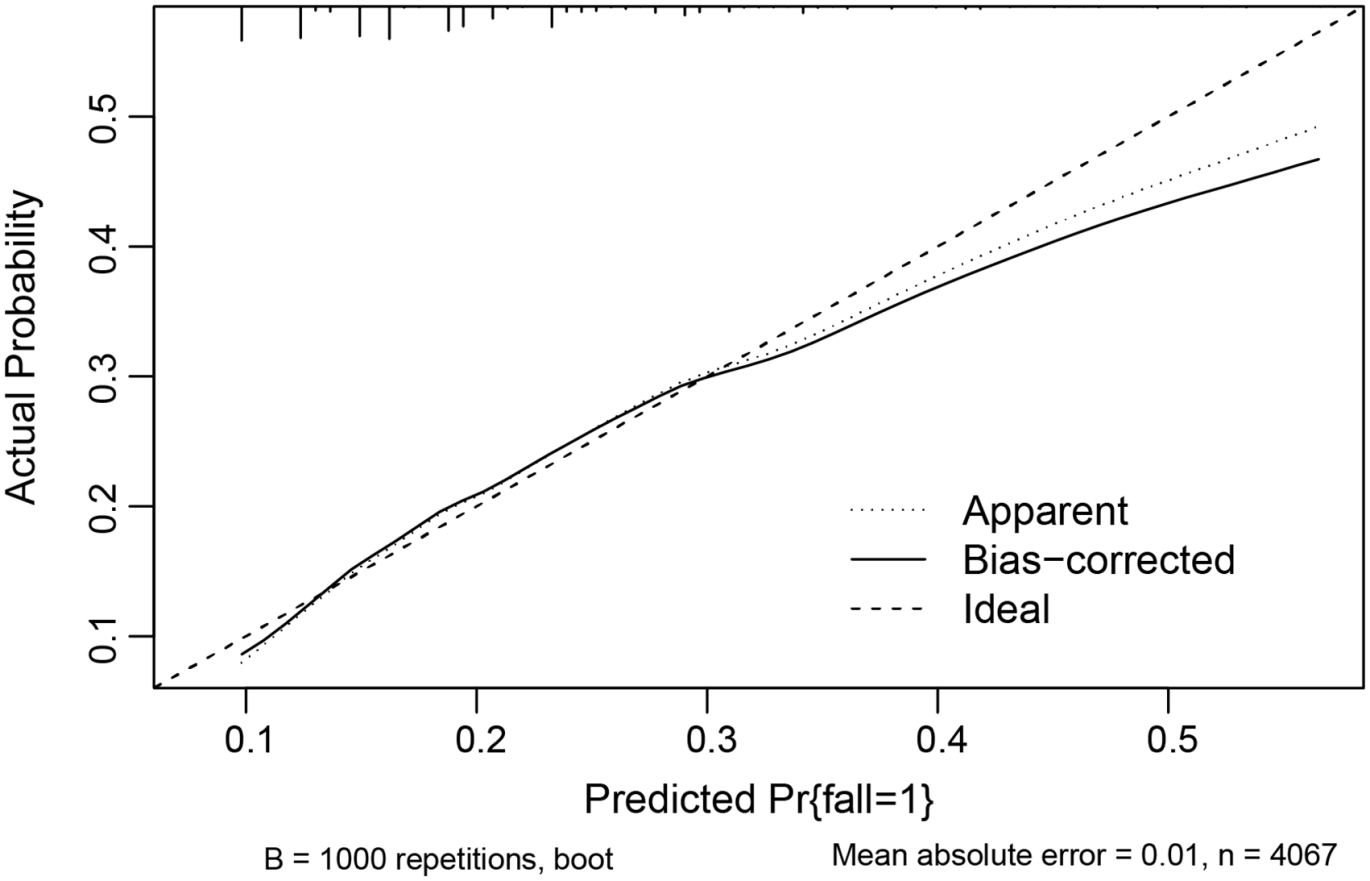
Calibration curve for fall prediction model.

As the above results showed that *CF* had a greater effect on fall risk than frailty alone, we further used *CF* as an independent factor to develop a fall prediction model. For convenience, age was set as a dichotomous variable with a cutoff value of 75 years. After adjusting for covariates in Model 3, sex, age (categorical), hearing difficulty, IADL disability, depressive status, anxiety status, serious illness diagnosed in the last 2 years, and *CF* were significantly correlated with falls. The OR (95%CI) for *CF* [1.87(1.07–3.25)] was the greatest among these factors (Figure 3). Next, a nomogram was constructed (Figure 4), which could be applied to calculate the risk of falling for community dwellers aged ≥60y. For example, for an 80-year-old woman with *CF*, CES-D score of 11, GAD-7 score of 13, IALD disability, and hearing loss but no serious illnesses in the past 2 years, the total score is 440. This score corresponds to a fall probability of 63%. The power of this prediction model was tested by ROC, which yielded an AUC of 0.646, with a sensitivity of 0.679 and a specificity of 0.532 (Figure 5). The C-index was 0.641. A calibration curve was drawn with the x-axis representing the predicted probability of falls and the y-axis representing the actual probability of falls (Figure 6).

## Discussion

The overall prevalence of falls and *CF* participants was 19.4% (790) and 1.4% (58), respectively. *CF* significantly increased the fall risk compared with the robust status and showed a cumulative effect on falls compared with CoI or frailty alone. Additionally, we extended these findings to develop a prediction model employing *CF*, sex, age, hearing difficulty, depression, anxiety, IADL disability, and serious illness in the past 2 years. This model had moderate discriminatory accuracy for falls. To the best of our knowledge, this is the first study to use *CF* to establish a risk evaluation model for fall prediction and presented a nomogram accordingly for practical use.

The *CF* prevalence varied in different studies, ranging from 1.0 to 50.1% (Qiu et al., 2022). This may be a result of different diagnostic criteria. According to the consensus for *CF* definition (Kelaiditi et al., 2013), several measurements for cognitive function and physical status are suggested. For cognitive function evaluation, our study employed the MMSE scale and observed a CoI prevalence lower than that (8.3 and 12.1%) in the other two studies using similar criteria (Brigola et al., 2019; Wang et al., 2022). This disparity may be due to a greater mean MMSE score of 28 in our participants than the score of 21 or 24 in the abovementioned studies. Since MMSE is highly influenced by socioeconomical factors (Jeong et al., 2007), better educational background (schooling year: 5.43 ± 4.79 vs. 3.54 ± 3.54) among the participants in our study could be the reason of the difference. This may also explain why, in another comparable aging study, the CoI prevalence of 22.0% using the HDS-R scale was higher than ours. The literacy rate in that study was 48.1%, much lower than that (75.0%) in our study. Another possible explanation would be that HDS-R covers more memory evaluation than MMSE does, which is highly relevant to the early manifestation of cognitive decline (Senda et al., 2020). For physical status evaluation, previous *CF* studies from Japan (Kim et al., 2019a), Italy (Solfrizzi et al., 2017), and the United States (Ge et al., 2021) utilized FP and found the frailty prevalence were 3.6, 3.1, and 5.6%, respectively. This prevalence was much lower than ours identified by FI method, suggesting that FI could diagnose more frail adults than FP does (Blodgett et al., 2015). Therefore, available assessments for *CF* differ in diagnostic efficacy. In view of increasing evidence on the associations between *CF* and adverse health outcomes, further studies proposing a uniform evaluation of *CF* are warranted.

Cognitive frailty (*CF*) showed a cumulative effect on falls than CoI or frailty on its own. As previously reported, Ge et al. (2021) discovered that *CF* participants had a 2.5-fold higher fall risk than their robust counterparts, whereas the fall risk among CoI and frail alone participants only increased by 22 and 53%, respectively. Contrary to this findings, Ma et al. (2021) found that frail adults had a 6.8-fold higher risk of falls than robust adults, while the OR for *CF* adults was 3.5. The underlying reason could be the different sample characteristics. The prevalence of falls among groups in Ge et al.’s study was as follows: 58.3% in the *CF* group, 45.2% in the frailty only group, 29.8% in the CoI only group, and 24.7% in the robust group. However, in Ma et al.’s study, the fall rate of 23.7% was the highest in the frailty only group. Our study results were consistent with the Ge et al.’s findings and demonstrated a greater fall rate in the *CF* group than in the other groups. Moreover, we observed that those with *CF* had poor MMSE and FI scores than did the other groups, which could partially explain the joint effect of *CF* on falls. This finding indicates that cognitive impairment may lead to a more severe physical decline and vice versa. With a poor functional status, the risks of adverse health outcomes increase, including falls (Martin et al., 2013; Aliberti et al., 2019; Chu et al., 2019). Thus, simultaneous assessments of frailty and cognitive function would be a better choice for predicting fall risk in the elderly (Ge et al., 2021).

Since *CF* may be a better index than CoI or frailty for fall risk assessment, we presented a prediction model employing *CF* as well as other fall-related factors. Similar to previous research findings (Ogliari et al., 2021; Lee Y. Y. et al., 2021; Luo et al., 2022; Nagarkar and Kulkarni, 2022), we found that sex, age, hearing difficulty, IADL disability, and depressive or anxious status were associated with falls. However, no association was found between falls and other formerly confirmed risk factors, such as sleep duration, vision impairment or BADL disability. This disparity may be due to different evaluation methods. For example, a previous study used a five-level rating scale for vision assessment, from “excellent” to “poor,” and found that visual impairment independently correlated with falls (Ogliari et al., 2021). Our study used a simpler assessment, and found no significant relationship between them. Furthermore, serious illness in the past 2 years was first reported in our study as a fall-associated factor, suggesting that a prolonged awareness of fall risk may be appropriate for those who have been critical ill in the past 2 years. As these factors can be easily acquired through questionnaires, our prediction model would be feasible for a large-scale screening for fall risk in the community.

The current study was the first to develop a fall prediction model based on *CF.* By analyzing a sample with an average age of 79 years, our findings may add to existing evidence and provide meaningful insights on fall risk prevention in this aging society. However, some limitations should be noted. First, because of a cross-sectional study design, our results did not demonstrate a causal relationship between *CF* and falls. We were only able to draw conclusions of association. Second, as most of the data in our study were self-reported and only dwellers without missing values were included, information and selection biases were inevitable. Third, other well-recognized fall risk factors were not included, such as fear of falling and recurrent falls. Finally, since the number of CoI group was only 7, relevant results should be interpreted with caution. Further studies including more relevant factors and larger sample would be more informative.

## Conclusion

In summary, this study observed that *CF* increased fall risk and had a cumulative effect on falls compared with CoI or frailty alone. Furthermore, a nomogram based on *CF*, age, sex, IADL disability, depression, anxiety, hearing difficulty, and a medical history of serious illness in the past 2 years yielded a moderate power of fall risk discrimination. Hence, joint assessments of cognitive function and frailty status may be beneficial for fall risk screening in communities. The nomogram based on *CF* could be a feasible prediction tool for this process.

## Data availability statement

The original contributions presented in the study are included in the article/Supplementary material, further inquiries can be directed to the corresponding author.

## Ethics statement

The studies involving human participants were reviewed and approved by the Ethical Committee of Peking University. The patients/participants provided their written informed consent to participate in this study.

## Author contributions

HC and LH: study concept, design, statistical analysis, and drafting the manuscript. YL: interpretation of data. YL and WX: interpretation of data and revision manuscript. J-WX: supervision the whole project. All authors contributed to the article and approved the submitted version.

## Funding

This work was supported by Guangxi Zhuang Autonomous Region Health and Family Planning Commission Self-Founded Scientific Research Project (Z20210496).

## Conflict of interest

The authors declare that the research was conducted in the absence of any commercial or financial relationships that could be construed as a potential conflict of interest.

## Publisher’s note

All claims expressed in this article are solely those of the authors and do not necessarily represent those of their affiliated organizations, or those of the publisher, the editors and the reviewers. Any product that may be evaluated in this article, or claim that may be made by its manufacturer, is not guaranteed or endorsed by the publisher.

## References

[ref1] Aliberti M. J. R. Cenzer I. S. Smith A. K. Lee S. J. Yaffe K. Covinsky K. E. (2019). Assessing risk for adverse outcomes in older adults: the need to include both physical frailty and cognition. J. Am. Geriatr. Soc. 67, 477–483. doi: 10.1111/jgs.15683, PMID: 30468258 PMC6510389

[ref2] Björgvinsson T. Kertz S. J. Bigda-Peyton J. S. McCoy K. L. Aderka I. M. (2013). Psychometric properties of the CES-D-10 in a psychiatric sample. Assessment 20, 429–436. doi: 10.1177/1073191113481998, PMID: 23513010

[ref3] Blodgett J. Theou O. Kirkland S. Andreou P. Rockwood K. (2015). Frailty in NHANES: comparing the frailty index and phenotype. Arch. Gerontol. Geriatr. 60, 464–470. doi: 10.1016/j.archger.2015.01.016, PMID: 25697060

[ref4] Brigola A. G. Ottaviani A. C. Alexandre T. Luchesi B. M. Pavarini S. (2019). Cumulative effects of cognitive impairment and frailty on functional decline, falls and hospitalization: a four-year follow-up study with older adults. Arch. Gerontol. Geriatr. 87:104005. doi: 10.1016/j.archger.2019.104005, PMID: 31901850

[ref5] Cesari M. Gambassi G. van Kan G. A. Vellas B. (2014). The frailty phenotype and the frailty index: different instruments for different purposes. Age Ageing 43, 10–12. doi: 10.1093/ageing/aft160, PMID: 24132852

[ref6] Chen Q. Tang B. Zhai Y. Chen Y. Jin Z. Han H.. (2020). Dynamic statistical model for predicting the risk of death among older Chinese people, using longitudinal repeated measures of the frailty index: a prospective cohort study. Age Ageing 49, 966–973. doi: 10.1093/ageing/afaa056, PMID: 32365173

[ref7] Chu N. M. Bandeen-Roche K. Tian J. Kasper J. D. Gross A. L. Carlson M. C.. (2019). Hierarchical development of frailty and cognitive impairment: clues into etiological pathways. J. Gerontol. A Biol. Sci. Med. Sci. 74, 1761–1770. doi: 10.1093/gerona/glz134, PMID: 31120105 PMC6777087

[ref8] Clegg A. Young J. Iliffe S. Rikkert M. O. Rockwood K. (2013). Frailty in elderly people. Lancet 381, 752–762. doi: 10.1016/s0140-6736(12)62167-9, PMID: 23395245 PMC4098658

[ref9] Florence C. S. Bergen G. Atherly A. Burns E. Stevens J. Drake C. (2018). Medical costs of fatal and nonfatal falls in older adults. J. Am. Geriatr. Soc. 66, 693–698. doi: 10.1111/jgs.15304, PMID: 29512120 PMC6089380

[ref10] Fried L. P. Tangen C. M. Walston J. Newman A. B. Hirsch C. Gottdiener J.. (2001). Frailty in older adults: evidence for a phenotype. J Gerontol. Biol. Sci. Med. Sci. 56, M146–M157. doi: 10.1093/gerona/56.3.M146 11253156

[ref11] Ganz D. A. Latham N. K. (2020). Prevention of falls in community-dwelling older adults. N. Engl. J. Med. 382, 734–743. doi: 10.1056/NEJMcp1903252 32074420

[ref12] Gauthier S. Reisberg B. Zaudig M. Petersen R. C. Ritchie K. Broich K.. (2006). Mild cognitive impairment. Lancet 367, 1262–1270. doi: 10.1016/S0140-6736(06)68542-5 16631882

[ref13] Ge M.-L. Simonsick E. M. Dong B.-R. Kasper J. D. Xue Q.-L. (2021). Frailty, with or without cognitive impairment, is a strong predictor of recurrent falls in a US population-representative sample of older adults. J. Gerontol. 76, e354–e360. doi: 10.1093/gerona/glab083, PMID: 33721909 PMC8562403

[ref14] Gordon E. H. Reid N. Khetani I. S. Hubbard R. E. (2021). How frail is frail? A systematic scoping review and synthesis of high impact studies. BMC Geriatr. 21:719. doi: 10.1186/s12877-021-02671-3, PMID: 34922490 PMC8684089

[ref15] Guo X. Pei J. Ma Y. Cui Y. Guo J. Wei Y.. (2022). Cognitive frailty as a predictor of future falls in older adults: a systematic review and meta-analysis. J. Am. Med. Dir. Assoc. doi: 10.1016/j.jamda.2022.10.011, PMID: 36423679

[ref16] Haddad Y. K. Bergen G. Florence C. S. (2019). Estimating the economic burden related to older adult falls by state. J. Public Health Manag. Pract. 25, E17–E24. doi: 10.1097/phh.0000000000000816, PMID: 29757813 PMC6314899

[ref17] Han Z. Shi-ge Q. Lu C. (2021). Prevalence of falls and fall-related injuries among Chinese community-dwelling older adults: a one-year retrospective follow-up data analysis. Chin. J. Public Health 37:1590. doi: 10.11847/zgggws1133277

[ref18] Immonen M. Haapea M. Simila H. Enwald H. Keranen N. Kangas M.. (2020). Association between chronic diseases and falls among a sample of older people in Finland. BMC Geriatr. 20:225. doi: 10.1186/s12877-020-01621-9, PMID: 32590946 PMC7318483

[ref19] Jeong J. W. Kim K. W. Lee D. Y. Lee S. B. Park J. H. Choi E. A.. (2007). A normative study of the revised Hasegawa dementia scale: comparison of demographic influences between the revised Hasegawa dementia scale and the mini-mental status examination. Dement. Geriatr. Cogn. Disord. 24, 288–293. doi: 10.1159/000107592, PMID: 17717415

[ref20] KelaiditiE.CesariM.CanevelliM.Abellan Van KanG.OussetP.-J.Gillette-GuyonnetS.. (2013). Cognitive frailty: rational and definition from an (IANA/IAGG) international consensus group. J. Nutr. Health Aging 17, 726–734. doi: 10.1007/s12603-013-0367-2, PMID: 24154642

[ref21] Kim H. Awata S. Watanabe Y. Kojima N. Osuka Y. Motokawa K.. (2019a). Cognitive frailty in community-dwelling older Japanese people: prevalence and its association with falls. Geriatr. Gerontol. Int. 19, 647–653. doi: 10.1111/ggi.13685, PMID: 31083795

[ref22] Lee Y. Y. Chen C. L. Lee I. C. Lee I. C. Chen N. C. (2021). History of falls, dementia, lower education levels, mobility limitations, and aging are risk factors for falls among the community-dwelling elderly: a cohort study. Int. J. Environ. Res. Public Health 18:9. doi: 10.3390/ijerph18179356, PMID: 34501947 PMC8430505

[ref23] Lee S. Chung J. H. Kim J. H. (2021). Association between sleep quality and falls: a Nationwide population-based study from South Korea. Int. J. Gen. Med. 14, 7423–7433. doi: 10.2147/IJGM.S331103, PMID: 34744453 PMC8566001

[ref24] Luo Y. Miyawaki C. E. Valimaki M. A. Tang S. Sun H. Liu M. (2022). Symptoms of anxiety and depression predicting fall-related outcomes among older Americans: a longitudinal study. BMC Geriatr. 22:749. doi: 10.1186/s12877-022-03406-8, PMID: 36100852 PMC9472405

[ref25] Ma Y. Li X. Pan Y. Zhao R. Wang X. Jiang X.. (2021). Cognitive frailty and falls in Chinese elderly people: a population-based longitudinal study. Eur. J. Neurol. 28, 381–388. doi: 10.1111/ene.14572, PMID: 33030300

[ref26] Ma L. Zhang L. Zhang Y. Li Y. Tang Z. Chan P. (2017). Cognitive frailty in China: results from China comprehensive geriatric assessment study. Front. Med. 4:174. doi: 10.3389/fmed.2017.00174, PMID: 29104866 PMC5655005

[ref27] Martin K. L. Blizzard L. Srikanth V. K. Wood A. Thomson R. Sanders L. M.. (2013). Cognitive function modifies the effect of physiological function on the risk of multiple falls--a population-based study. J. Gerontol. A Biol. Sci. Med. Sci. 68, 1091–1097. doi: 10.1093/gerona/glt010 23410920

[ref28] Montero-Odasso M. Speechley M. (2018). Falls in cognitively impaired older adults: implications for risk assessment and prevention. J. Am. Geriatr. Soc. 66, 367–375. doi: 10.1111/jgs.15219, PMID: 29318592

[ref29] Moreira N. B. Rodacki A. L. F. Pereira G. Bento P. C. B. (2018). Does functional capacity, fall risk awareness and physical activity level predict falls in older adults in different age groups? Arch. Gerontol. Geriatr. 77, 57–63. doi: 10.1016/j.archger.2018.04.002, PMID: 29673964

[ref30] Moreland B. Kakara R. Henry A. (2020). Trends in nonfatal falls and fall-related injuries among adults aged ≥ 65 years—United States, 2012–2018. Morb. Mortal. Wkly Rep. 69, 875–881. doi: 10.15585/mmwr.mm6927a5, PMID: 32644982 PMC7732363

[ref31] Muir S. W. Karen G. Montero O. M. M. (2012). The role of cognitive impairment in fall risk among older adults: a systematic review and meta-analysis. Age Ageing 41, 299–308. doi: 10.1093/ageing/afs012, PMID: 22374645

[ref32] Nagarkar A. Kulkarni S. (2022). Association between daily activities and fall in older adults: an analysis of longitudinal ageing study in India (2017-18). BMC Geriatr. 22:203. doi: 10.1186/s12877-022-02879-x, PMID: 35287596 PMC8922744

[ref33] Ogliari G. Ryg J. Qureshi N. Andersen-Ranberg K. Scheel-Hincke L. L. Masud T. (2021). Subjective vision and hearing impairment and falls among community-dwelling adults: a prospective study in the survey of health, ageing and retirement in Europe (SHARE). Eur. Geriatr. Med. 12, 1031–1043. doi: 10.1007/s41999-021-00505-4, PMID: 34003480

[ref34] Plummer F. Manea L. Trepel D. McMillan D. (2016). Screening for anxiety disorders with the GAD-7 and GAD-2: a systematic review and diagnostic metaanalysis. Gen. Hosp. Psychiatry 39, 24–31. doi: 10.1016/j.genhosppsych.2015.11.005, PMID: 26719105

[ref35] Qiu Y. Li G. Wang X. Zheng L. Wang C. Wang C.. (2022). Prevalence of cognitive frailty among community-dwelling older adults: a systematic review and meta-analysis. Int. J. Nurs. Stud. 125:104112. doi: 10.1016/j.ijnurstu.2021.104112, PMID: 34758429

[ref36] Rivan N. F. M. Singh D. K. A. Shahar S. Wen G. J. Rajab N. F. Din N. C.. (2021). Cognitive frailty is a robust predictor of falls, injuries, and disability among community-dwelling older adults. BMC Geriatr. 21:593. doi: 10.1186/s12877-021-02525-y, PMID: 34696720 PMC8543922

[ref37] Rockwood K. Andrew M. Mitnitski A. (2007). A comparison of two approaches to measuring frailty in elderly people. J. Gerontol.-Biol. Sci. Med. Sci. 62, 738–743. doi: 10.1093/gerona/62.7.738, PMID: 17634321

[ref38] Searle S. D. Mitnitski A. Gahbauer E. A. Gill T. M. Rockwood K. (2008). A standard procedure for creating a frailty index. BMC Geriatr. 8:24. doi: 10.1186/1471-2318-8-24, PMID: 18826625 PMC2573877

[ref39] Senda M. Terada S. Takenoshita S. Hayashi S. Yabe M. Imai N.. (2020). Diagnostic utility of the Addenbrooke's cognitive examination-III (ACE-III), mini-ACE, mini-mental state examination, Montreal cognitive assessment, and Hasegawa dementia scale-revised for detecting mild cognitive impairment and dementia. Psychogeriatrics 20, 156–162. doi: 10.1111/psyg.12480, PMID: 31448862

[ref40] Solfrizzi V. Scafato E. Lozupone M. Seripa D. Giannini M. Sardone R.. (2017). Additive role of a potentially reversible cognitive frailty model and inflammatory state on the risk of disability: the Italian longitudinal study on aging. Am. J. Geriatr. Psychiatry 25, 1236–1248. doi: 10.1016/j.jagp.2017.05.018, PMID: 28689645

[ref41] Tsai Y. J. Yang P. Y. Yang Y. C. Lin M. R. Wang Y. W. (2020). Prevalence and risk factors of falls among community-dwelling older people: results from three consecutive waves of the national health interview survey in Taiwan. BMC Geriatr. 20:529. doi: 10.1186/s12877-020-01922-z, PMID: 33297968 PMC7724833

[ref42] Wang C. Chong Y. Wang L. Wang Y. (2022). The correlation between falls and cognitive frailty in elderly individuals with hypertension in a Chinese community. Front. Aging Neurosci. 14:783461. doi: 10.3389/fnagi.2022.783461 35645780 PMC9131718

[ref43] Zeng Y. Poston D. L. Vlosky D. A. Gu D. (2008). Healthy Longevity in China: Demographic, Socioeconomic, and Psychological Dimensions. Springer: Netherlands.

[ref44] Zhang Y. Xiong Y. Yu Q. Shen S. Chen L. Lei X. (2021). The activity of daily living (ADL) subgroups and health impairment among Chinese elderly: a latent profile analysis. BMC Geriatr. 21:30. doi: 10.1186/s12877-020-01986-x, PMID: 33413125 PMC7791986

